# Withdrawal-induced delirium associated with a benzodiazepine switch: a case report

**DOI:** 10.1186/1752-1947-5-207

**Published:** 2011-05-26

**Authors:** Herbert Bosshart

**Affiliations:** 1 ARUD, Group of Private Outpatient Facilities for the Treatment of Substance Use and Co-occurring Disorders, Sihlhallenstrasse 30, CH-8026 Zurich, Switzerland

## Abstract

**Introduction:**

Introduced in the early 1960s, diazepam remains among the most frequently prescribed benzodiazepine-type sedatives and hypnotics. Patients with chronic use of short-acting benzodiazepines are frequently switched to diazepam because the accumulating, long-acting metabolite, N-desmethyl-diazepam, prevents benzodiazepine-associated withdrawal symptoms, which can occur during trough plasma levels of short-acting benzodiazepines. Although mild to moderate withdrawal symptoms are frequently observed during benzodiazepine switching to diazepam, severe medical complications associated with this treatment approach have thus far not been reported.

**Case presentation:**

A 64-year-old female Caucasian with major depression, alcohol dependence and benzodiazepine dependence was successfully treated for depression and, after lorazepam-assisted alcohol detoxification, was switched from lorazepam to diazepam to facilitate benzodiazepine discontinuation. Subsequent to the benzodiazepine switch, our patient unexpectedly developed an acute delirious state, which quickly remitted after re-administration of lorazepam. A newly diagnosed early form of mixed dementia, combining both vascular and Alzheimer-type lesions, was found as a likely contributing factor for the observed vulnerability to benzodiazepine-induced withdrawal symptoms.

**Conclusion:**

Chronic use of benzodiazepines is common in the elderly and a switch to diazepam often precedes benzodiazepine discontinuation trials. However, contrary to common clinical practice, benzodiazepine switching to diazepam may require cross-titration with slow tapering of the first benzodiazepine to allow for the build-up of N-desmethyl-diazepam, in order to safely prevent severe withdrawal symptoms. Alternatively, long-term treatment with low doses of benzodiazepines may be considered, especially in elderly patients with chronic use of benzodiazepines and proven vulnerability to benzodiazepine-associated withdrawal symptoms.

## Introduction

The benzodiazepine (BZD)-type sedatives and hypnotics, introduced in the 1960s, marked a major advance in the treatment of anxiety, depression, insomnia and seizures, not least because of their improved therapeutic index. The first BZDs marketed by F. Hoffmann-La Roche, chlordiazepoxide (Librium^®^) and diazepam (Valium^®^), became immensely popular. Diazepam was the most widely prescribed drug in the United States and Europe for nearly two decades. Even with the subsequent introduction of numerous other BZDs, diazepam remained one of the first-choice BZDs among prescribers and in 1985 was added to the World Health Organization's essential medicines list [1] for its anti-convulsant, anxiolytic, sedative-hypnotic and pre-medicant uses. In 2009, lorazepam was added to this list and is now recommended as an alternative to diazepam, notably for its anti-convulsant properties. The superior efficacy of lorazepam over diazepam in the treatment of status epilepticus, as demonstrated by Alldredge *et al*. nearly one decade ago [2], is explained, at least in part, by the pharmacokinetic properties of both drugs, which were described in detail in the 1980s by Greenblatt *et al*. [3,4].

BZD-associated withdrawal symptoms have been recognized for as long as BZDs have been in use. Over the years, it has become abundantly clear that chronic use of BZDs results in tolerance, rebound phenomena and dependence, making it difficult for patients to discontinue BZD use. The two basic treatment options available to patients with chronic BZD use are (i) BZD discontinuation or (ii) low-dose BZD maintenance. Where BZD discontinuation is favored, a frequently employed BZD detoxification strategy is to switch to diazepam, or to other BZDs with long elimination half-lives of the parent drug or its active metabolites, followed by slow tapering of the drug. In individuals with chronic BZD use, a BZD switch to diazepam [5] frequently results in mild or moderate withdrawal symptoms, such as distortions in perception, mood and cognition or disturbances in sensory and motor functions. However, severe medical complications, such as seizures or delirium, which are known to occur relatively infrequently, even after abrupt BZD discontinuation, have not been associated with a transfer to diazepam. In particular, withdrawal-induced delirium associated with a switch from lorazepam to diazepam has thus far not been reported. Interestingly, however, Onyett pointed out as early as 1989 that, in some cases, switching patients from lorazepam to diazepam may require cross-titration [6]. Perhaps the only report describing a similar case was published by Zipursky *et al*. [7]. The authors described a 68-year-old patient with alprazolam withdrawal delirium unresponsive to treatment with diazepam but responsive to treatment with alprazolam [7].

In this report we describe the case of an elderly woman with a number of mental and physical conditions who developed an acute delirious state after being switched from lorazepam to diazepam. Drug pharmacokinetics and psychiatric vulnerabilities are considered as possible causal factors.

## Case presentation

A 64-year-old Caucasian woman with major depression (Diagnostic and Statistical Manual of Mental Disorders-Text Revision (DSM IV-TR): 296.33), alcohol dependence (DSM IV-TR: 303.90) and benzodiazepine (lorazepam) dependence (DSM IV-TR: 304.10) was referred to a specialist ward for the treatment of substance use and co-occurring mental disorders. Daily use of alcohol started ten years prior to admission and daily use of lorazepam one year prior. In addition, our patient had a history of medical conditions for which she had been receiving medication. Levothyroxine was used to treat hypothyroidism (thyroxine-stimulating hormone < 0.01 mU/L; free thyroxine = 20.9 pmol/L, free tri-iodothyronine = 8.5 pmol/L), standard female hormone replacement therapy consisting of 1 mg 17β-estradiol and 5 mg dydrogesterone was used to alleviate and prevent symptoms associated with menopause, and atorvastatin was prescribed to control hyperlipidemia. Felodipine, metoprolol and lisinopril were used to treat hypertension. Salicylate was used for the prevention of cardiac infarction. Pantoprazole was used to alleviate symptoms related to gastro-esophageal reflux. All prescriptions, including a previous trial with the antidepressant mianserin, were well tolerated without significant side effects and, except for mianserin, were continued after admission without dose changes.

On admission, lorazepam, at 5-10 mg daily, was begun to prevent alcohol- and BZD-associated withdrawal symptoms. Antidepressant treatment with mianserin (60 mg daily) resulted in a partial response. A three-week add-on trial with venlafaxine (maximum dose 450 mg daily) resulted in further improvement of depressive symptoms. However, residual depressive symptoms persisted and mianserin was switched to mirtazapine (maximum dose 75 mg daily). Complete remission was achieved two months later and venlafaxine maintained at 150 mg daily and mirtazapine at 60 mg daily (relapse prevention).

Lorazepam was tapered and discontinued after six weeks of treatment. One day after the last lorazepam dose, our patient exhibited difficulties in maintaining attention and showed signs of psychomotor agitation, anxiety and disturbed thought processes, such as loosened, illogical and tangential associations. Dissociative symptoms were also observed. These signs were consistent with an ensuing BZD withdrawal syndrome (DSM IV-TR: 292.0) and suggested that an even more protracted discontinuation phase may have been necessary. Delirium was not diagnosed at this time because orientation remained intact and the severity of the symptoms was not in excess of those usually associated with a BZD withdrawal syndrome. Lorazepam was resumed at 3 mg per day. Symptoms of withdrawal resolved and lorazepam was maintained at 3 mg daily for one month. Lorazepam was then discontinued without tapering, and diazepam was started at an equivalent dose of 15 mg daily. Less than one day after the BZD switch, our patient became increasingly irritable and agitated and eventually exhibited fluctuating levels of consciousness with reduced clarity of awareness and reduced ability to maintain attention. Disorientation, incomprehensible speech and memory deficits were also noted. The diagnosis of a BZD intoxication delirium was ruled out based on the absence of slurred speech, incoordination, unsteady gait, nystagmus or stupor. Our patient was diagnosed with BZD withdrawal delirium (DSM IV-TR: 292.81) and received 4 mg of lorazepam combined with 4 mg of haloperidol. The acute delirious state resolved within hours and our patient remained well. Haloperidol was stopped after two weeks. Lorazepam was continued at 4 mg daily. Our patient was dismissed one month later with a lorazepam maintenance dose of 4 mg per day.

An initial review of the prescriptions and the diagnostic tests performed in the course of treatment failed to reveal our patient's vulnerabilities to delirious states. Except for a slight increase of plasma diazepam levels in the presence of metoprolol, no relevant drug interactions were identified. A physical examination, laboratory workup and an analysis of cerebrospinal fluid were unremarkable. In particular, there was no evidence of hepatic dysfunction. Serum levels of alanine and aspartate aminotransferase, alkaline phosphatase, lactate dehydrogenase, and bilirubin were normal. Prothrombin time was within the normal range. Serum γ-glutamyltransferase was slightly elevated (74 U/L; normal range, < 55 U/L). However, serum concentrations of O-desmethyl-venlafaxine (a liver-derived metabolite of venlafaxine) were seven-fold higher than those of venlafaxine. Taken together, these results suggested normal liver function with intact hepatic drug metabolism and ruled out the differential diagnosis of a diazepam-exacerbated hepatic encephalopathy.

Electroencephalogram recordings showed the rhythm of alpha waves (11-12 Hz) and beta waves (18-22 Hz) to be as expected during treatment with lorazepam. Epileptiform discharges or focal abnormalities were not present. Electrocardiogram recordings showed a normal sinus rhythm with a frequency of around 90 per minute. Neuropsychological tests revealed only mild cognitive deficits. At the time these test were performed our patient was receiving the medication to treat her physical conditions as described above. In addition, she received mirtazapine (60 mg daily), venlafaxine (150 mg daily) and lorazepam (4 mg daily). The Mini-Mental State Examination score was 27. The Consortium to Establish a Registry for Alzheimer's Disease test revealed minimal cognitive impairment, mainly affecting her verbal memory (left hippocampus system), executive functions (frontal lobe) and mental rotation (parietal lobe). On the Hamburg Wechsler Intelligence test for adults, she achieved 90 points, a score slightly below average.

Computed tomography scans showed no signs of brain atrophy. However, using T2 pulses, magnetic resonance imaging showed scattered sub-cortical signal disturbances in her frontal, parietal and occipital regions. Additional signal disturbances were found in her pontine regions and brain stem. Together, these findings were consistent with sub-cortical atherosclerotic encephalopathy, in other words, Binswanger's disease. Positron emission tomography scanning of the brain using the radio-labeled glucose analog ^18^F-fluorodeoxyglucose (FDG) showed diminished FDG uptake in temporal and parietal cortical regions. Lower FDG accumulation was also found in parts of the visual cortex and in both her basal ganglia and thalamus. The absence of hallucinations, Parkinsonian or extra-pyramidal symptoms, together with the previously well-tolerated trial with haloperidol, argued against Lewy body dementia [8]. The most likely diagnosis was an early form of mixed dementia, combining both vascular (DSM IV-TR: 290.40) and Alzheimer-type lesions (DSM IV-TR: 294.10). Thus, the neuro-imaging results suggested that our patient might be liable to develop a delirious state in response to chemically induced brain disturbances. Before leaving our hospital, our patient was started on donepezil, shown to improve cognition in Alzheimer's disease [9].

## Discussion

In agreement with the present case, evidence now suggests that (i) both short-and long-term BZD use is associated with old age, female sex, psychological stress and physical disease [10], (ii) long-term BZD use in old age is typically associated with mood disorders, alcohol abuse and female sex [11] and (iii) depression in old age is associated with the use of alcohol and prescription drugs, with female sex and with medical conditions such as heart disease and Alzheimer's disease [12].

Since chronic BZD use constitutes a risk for cognitive decline [13] and since our patient was diagnosed with an early form of dementia, exhibiting mild cognitive impairments, the treatment goal was to discontinue BZD use. However, since tapering lorazepam resulted in unacceptable withdrawal symptoms, a switch to diazepam was considered. At first glance, the observation that switching lorazepam to an equivalent dose of diazepam resulted in a withdrawal-induced delirious state is puzzling because BZD withdrawal delirium is usually associated with sudden discontinuation from short-acting BZDs [14], and long-acting BZDs or long-acting BZD metabolites are usually associated with intoxication-induced but not withdrawal-induced delirium [14]. Generally, however, long-acting BZDs, particularly at high doses, are frequently associated with delirium and commonly contribute to cognitive impairment in dementia [14]. An interesting explanation is offered by Greenblatt *et al*. who found that, despite its longer half-life, unbound diazepam distributes more extensively into tissue than lorazepam does [3] and thus has a shorter duration of action than lorazepam. Additionally, as shown in animals, diazepam brain-to-plasma ratios decrease rapidly within minutes [4]. Lorazepam, in contrast, shows a more sustained build-up in the central nervous system [4]. Consequently, switching from lorazepam to diazepam may lead to withdrawal symptoms even when equivalent doses are used.

While our case demonstrates a particular vulnerability to BZD-induced withdrawal symptoms in a patient with discrete vascular and Alzheimer-type lesions, the molecular mechanisms responsible for this vulnerability remain unexplained. However, irrespective of the presence of vascular or Alzheimer-type lesions, which may be associated with such vulnerability, the complex nature of the γ-amino-butyric acid (GABA) system, through which BZDs mediate their anxiolytic, sedative, anti-convulsant and muscle relaxant effects, may provide other clues.

GABA, the major inhibitory neurotransmitter in the mammalian central nervous system, mediates fast post-synaptic inhibition through binding to the GABA-A receptor, a hetero-pentameric chloride-selective ligand-gated ion channel [15]. To date, 19 different types of GABA-A polypeptide chains (α1-α6, β1-β3, γ1-γ3, δ, ε, θ, π, ρ1-ρ3) have been characterized [16]. Many of the theoretically possible pentamers are not expressed at the cell surface. Nevertheless, an impressive number of different GABA-A pentamers are found with different distributions in the mammalian brain. The most abundant subtypes are 2α1-2β2-1γ2, 2α2-2β3-1γ2 and 2α3-2β3-1γ2 [16].

The observed vulnerability to BZD-induced withdrawal symptoms could be explained by the expression patterns of GABA-A pentamers in our patient's brain, because different BZD effects are mediated by different GABA-A pentamers [17]. Furthermore, the assumption that the most extensively investigated prototypic agonist, diazepam, exerts identical allosteric effects on GABA-A receptors as other BZDs, lorazepam for example, may not be true. These considerations leave open the possibilities that inborn differences in the patient's GABA system, BZD-induced changes in this system [18] or changes in the GABA system as a result of the assumed mixed vascular and Alzheimer-type dementia may have contributed to a special vulnerability to BZD-induced withdrawal symptoms. Both clinical and basic research is needed to support or dismiss these ideas as possible mechanisms.

## Conclusion

This case report describes a 64-year-old female Caucasian with several pre-existing medical conditions and psychiatric disorders common for her age. These were treated successfully with one notable exception, BZD dependence, for which discontinuation was the favored treatment goal. Unexpectedly, our patient developed a severe adverse reaction (delirium) associated with a switch from lorazepam to diazepam.

Since BZD prescriptions in the elderly are common to almost all medical subspecialties, severe adverse events associated with BZD use must be reported quickly to alert prescribers and to improve treatment safety and quality. The present case suggests that elderly polymorbid patients with chronic BZD use may benefit from cross-tapering when switched to diazepam. Finally, long-term treatment with low doses of BZDs may be considered in these patients when BZD discontinuation trials fail.

## Consent

Written informed consent was obtained from the patient for publication of this case report. A copy of the written consent is available for review by the Editor-in-Chief of this journal.

## Competing interests

The author declares that they have no competing interests.

## Author's information

The author is a board-certified psychiatrist and head of a private outpatient facility for the treatment of substance use and co-occurring disorders. The author has a background in molecular and cell biology research.
